# Enhancing Psychiatry Resident Bonding and Education using an Escape Room Challenge Activity

**DOI:** 10.1192/j.eurpsy.2024.1656

**Published:** 2024-08-27

**Authors:** G. Lee, G. Russell, H. Farhan, J. Lin, L. Spring

**Affiliations:** Psychiatry, Stony Brook University Hospital, Stony Brook, United States

## Abstract

**Introduction:**

An “escape room” is a game requiring teamwork and problem-solving during which a series of puzzles are solved to escape a locked room. Various escape room activities have been designed for healthcare professionals, including internal medicine residents and nursing students (Anderson *et al*. Simulation & Gaming 2021; 52(1) 7-17; Rodríguez-Ferrer *et al*. BMC Med Educ 2022; 22:901; Khanna *et al.* Cureus 2021; 13 (9) e18314). Escape rooms provide an opportunity for social activity, an important component of resident wellness (Mari *et al*. BMC Med Educ 2019; 19(1):437). This abstract describes an escape room challenge designed and implemented at our psychiatry residency program quarterly wellness afternoon event, which is an afternoon session dedicated to resident wellness.

**Objectives:**

The objective of this project was to design and implement an escape room challenge containing multiple game mechanics, including hidden roles, information asymmetry, acting, logical deduction, and spying. This activity was conducted to enhance bonding among residents while reinforcing knowledge in psychiatry.

**Methods:**

We designed and implemented an escape room for 22 residents. Residents were divided into four teams each tasked with completing a sequence of puzzles to open the final lockbox. Two novel mechanics were added to the activity. Each team had a “clue holder” with clues to help solve all the puzzles. This team member had to conceal their identity because, if any of the other teams identified this person, the original winning team would have to give up the prize to the team that guessed the identity of this person. One member of each team was assigned a “spy” role whose mission was to make it hard for the clue holder to reveal all the clues. An anonymous post-activity survey was completed using Google Forms.

**Results:**

The script was set in a fictional, abandoned psychiatric emergency room. The first task was a visual puzzle of a historic figure in psychiatry. The second activity involved residents guessing the psychotropic medication being acted out by another resident in the style of charades. The third activity required residents to apply developmental milestones to decode a combination lock. The fourth puzzle involved residents solving riddles by using information gathered from resident profiles on the residency program website.

Eleven (50%) residents completed the post-game survey. All residents answered true or very true that they enjoyed the game and that participation helped them better connect with their peers. Eight (73%) residents answered true or very true that they learned something from the activity.

**Conclusions:**

An adapted escape room challenge is a novel wellness activity that enhance resident collegiality, teamwork, and bonding. All residents who completed the post-activity survey indicated that they enjoyed the activity and felt more connected to their peers afterwards.

**Disclosure of Interest:**

None Declared

